# Parents’ and neonatal healthcare professionals’ views on barriers and facilitators to parental presence in the neonatal unit: a qualitative study

**DOI:** 10.1186/s12887-024-04758-3

**Published:** 2024-04-24

**Authors:** Stephanie Vanessa Schmid, Christine Arnold, Sophie Jaisli, Benedikt Bubl, Erika Harju, André Kidszun

**Affiliations:** 1 Division of Neonatology, Department of Pediatrics, Inselspital, Bern University Hospital, University of Bern, Friedbühlstrasse 19, Bern, 3010 Switzerland; 2 Faculty of Health Sciences and Medicine, University of Lucerne, Alpenquai 4, Lucerne, 6002 Switzerland; 3 School of Health Sciences, ZHAW Zurich University of Applied Sciences, Katharina-Sulzer-Platz 9, Winterthur, 8401 Switzerland

**Keywords:** Parental presence, Preterm infant, Parents, Neonatal unit, Barriers, Facilitators

## Abstract

**Background:**

Parent and infant separation in the neonatal unit is associated with adverse health outcomes. Family-integrated care has several advantages and the potential to reduce these adverse outcomes but requires parental presence. This study aimed to explore the views of parents and neonatal healthcare professionals (nHCPs) on barriers and facilitators to parental presence in a Swiss neonatal unit and to identify possible differences between nHCPs and parents, and between mothers and fathers.

**Methods:**

Data were collected through semi-structured interviews with parents and focus group discussions with nHCPs. Inductive content analysis was used to identify barriers and facilitators to parental presence in the neonatal unit.

**Results:**

Twenty parents (10 mothers and 10 fathers) and 21 nHCPs (10 nurses and 11 physicians) participated in the study. Parents and nHCPs experienced barriers and facilitators related to: (1) Structural factors of the institution, such as infrastructure or travel and distance to the neonatal unit. (2) Organization and time management of parental presence, daily activities, and work. (3) Resources, which include factors related to the legal situation, support services, family, and friends. (4) Physical and psychological aspects, such as pain, which mainly affected mothers, and aspects of emotional distress, which affected both parents. Self-care was an important physical and psychological facilitator. (5) Parent-professional interaction. Parental presence was influenced by communication, relationship, and interaction in infant care; and (6) Cultural aspects and language. Some perspectives differed between mothers and fathers, while the overall views of parents and nHCPs provided complementary rather than conflicting insights. Using visit plans to support the organization, educating nHCPs in knowledge skills and available resources to improve encouragement and information to parents, strengthening parent self-care, and improving nHCPs’ attitudes towards parental presence were seen as possible improvements.

**Conclusions:**

Multifactorial barriers and facilitators determine parental presence and experience in the neonatal unit. Parents and nHCPs made specific recommendations to improve parental presence.

**Supplementary Information:**

The online version contains supplementary material available at 10.1186/s12887-024-04758-3.

## Background

The neonatal unit provides essential medical treatment and care for preterm infants and sick newborns. In-hospital stay is often characterized by parent-infant separation, making the hospitalization period extremely stressful and demanding for parents and infants [1–5]. Parents who have an infant in the neonatal unit may experience psychological issues such as stress, anxiety, sadness, fatigue, self-blame, less positive feelings toward their infant, or other depressive symptoms [2, 3, 6, 7]. Family Integrated Care (FICare) and parent-infant closeness can reduce parental stress and anxiety while improving parents’ well-being, mental health, self-efficacy, and relationship with their infants [8–13]. FICare further positively impacts infant weight gain, brain development, and breastfeeding outcomes [9, 10, 12, 13]. Kangaroo Mother Care (KMC) with skin-to-skin contact as a part of FICare is considered the most effective way to strengthen parent-infant bonding, build parents’ resilience as competent mothers and fathers, and provide immediate and long-term benefits for both parents and infants [14–19]. In particular, skin-to-skin contact stabilizes the infant’s heart and respiratory rate, improves thermoregulation and oxygen saturation, reduces nosocomial infections and has an overall positive effect on the risk of mortality and length of hospital stay [18–24]. Despite knowledge of the many benefits of FICare, closeness, and KMC, parent-infant separation is still common in neonatal units [5, 25, 26]. The promotion of FICare, closeness, and KMC is implemented to varying degrees, and their implementation faces various barriers and challenges [5, 27–34]. Mixed evidence exists regarding predictors of parental presence. The presence of siblings and increasing distance from home to the hospital are the most common barriers to parental presence [26, 35, 36]. Other influencing factors were found to be room type, medical status such as surgical history or neurological comorbidity, restrictive visitation policies, family support, and overnight accommodations [26, 35–37]. Available studies on barriers and facilitators to FICare interventions, predictors of parental presence, and parental experiences suggest that mothers and fathers may be affected by different factors. Previous studies have mainly included nurses rather than physicians and have shown complementary aspects to those reported by parents [34, 36]. Overall, there is insufficient knowledge about the barriers to facilitating parental presence in the neonatal unit, especially with regard to possible geographical or cultural differences.

This study aimed to explore the views of parents and neonatal healthcare professionals (nHCPs) on barriers and facilitators to parental presence in a Swiss neonatal unit and to identify possible differences between nHCPs and parents, and between mothers and fathers.

## Methods

### Study design and setting

A qualitative research approach was applied to obtain rich and in-depth data [38]. For this qualitative analysis we applied an inductive approach, starting from the data and analyzing themes and patterns according to Mayring [39]. Our study was conducted at the Division of Neonatology, Department of Pediatrics, at the Inselspital Bern in Switzerland, which cares for approximately 700 infants yearly. The neonatal unit consists of three wards, the neonatal intensive care unit, and two intermediate care units. This neonatal unit has a wide catchment area, up to 150 km in the case of the sample. In addition to infants born in house, infants from various hospitals in the canton of Bern are also transferred to the neonatal unit.

### Participants and recruitment

We recruited 20 parents (10 mothers and 10 fathers) and 21 nHCPs (10 nurses and 11 physicians). The sampling was based on a purposive approach to obtain “information-rich” data and an in-depth understanding. Non-probability sampling enables gathering qualitative responses, which leads to better insights [40]. Participants were healthy (self-reported), non-bereaved, German-speaking parents whose infant was hospitalized for at least 14 days to participate. Thus, a heterogeneous sample in terms of gestational age, length of hospital stay, number of children, and singletons or twins was considered. Physicians and nurses with at least 12 months of clinical experience were recruited. Parent recruitment was conducted face-to-face, and nHCPs were asked to participate during morning reports in July and August 2022 by the principal investigator S.S.

### Data collection

Semi-structured interviews were conducted with parents only and focus group discussions (FGDs) with nHCPs (nurses and physicians) by S.S. who is experienced in qualitative research. She interviewed mothers and fathers separately, and nurses and physicians were divided into separate discussion groups. The composition of the FGDs was chosen to minimize the group effect due to status and background. The interviews and FGDs were held based on two different interview guides *(see Additional file 1 and 2)* specifically created to meet the objectives of the study. We conducted a pilot test with an obstetrician and a scientist to revise and finalize the interview guides. To achieve breadth of coverage of all topics of interest and depth of intended content in each question, we used open-ended, non-leading, and probing questions. Follow-up questions were used to address key dimensions of the topic. Interviews were closed with a debriefing to avoid missing important points to the respondent [40, 41].

The interviews and discussions took place in the neonatal unit in a room separate from the patient rooms. Since the interviewer’s (S.S.) first contact with this neonatal unit was for this study as part of a research internship, she met the participants for the first time through recruitment and introduced her profession and function. All interviews were audio-recorded and conducted in Swiss German or German (the native language of the participant). The audio files were transcribed and translated from Swiss German into German by S.S. using MAXQDA software (Release 22.2.0, VERBI GmbH, Berlin, Germany) based on previously defined transcription rules. The transcripts were not returned to the participants for comment due to time constraints and the decision not to use parental time resources.

### Data analysis

Mayring’s inductive content analysis was used to identify barriers and facilitators to parental presence across the interviews and FGDs. The analysis approach is systematic and intersubjectively verifiable, but still does justice to the complexity and the need for interpretation of the source material [39]. Two researchers (S.S., C.A.) coded the data. Initially, open coding was used, and a preliminary coding system was developed derived from the data, which was expanded and adapted during the coding process. The two researchers regularly discussed and derived categories to ensure intercoder congruity and to achieve consensus. After coding, the anchor quotes were translated from German into English by S.S. and reviewed by a professional translator.

## Results

### Parents’ characteristics

Between July to September 2022, 20 parents participated. Parent’s characteristics are summarized in Table 1. The interviews ranged between 10 and 30 min with an average time of 23 min. The infants of the enrolled parents were born between 25^3/7^ and 34^6/7^ weeks of gestation and were hospitalized for an average of 37 days with a range of 14 to 78 days at the interview date.

**Table 1:** Parents’ characteristics

Characteristics	n (%) N = 20
**Age** in years; *mean (SD), range*	32.2 (± 5.7); 24–49
**Gender**	
Female	10 (50)
Male	10 (50)
**Nationality**	
Swiss only	18 (90)
Other	2 (10)
**Pregnancy**	
Singleton	14 (70)
Twins	6 (30)
**Birth mode**	
Cesarean section	19 (95)
Natural birth	1 (5)
**Number of children** (incl. newborn(s))	
1	10 (50)
2	8 (40)
3	1 (5)
4	1 (5)

SD = standard deviation

### Neonatal healthcare professionals’ characteristics

In total, 21 nHCPs (10 nurses and 11 physicians) participated in four FGDs. The FGDs ranged between 28 and 32 min, with an average of 30 min. Neonatal healthcare professionals’ characteristics are summarized in Table 2.

**Table 2:** Neonatal healthcare professionals’ characteristics

Characteristics	n (%) N = 21
**Age** in years; *mean (SD), range*	38.6 (± 12.1), 23–63
**Gender**	
Female	17 (81)
Male	4 (19)
**Profession**	
Nurse	10 (48)
Physician	11 (52)

SD = standard deviation

### Barriers and facilitators to parental presence

**Fig. 1:**
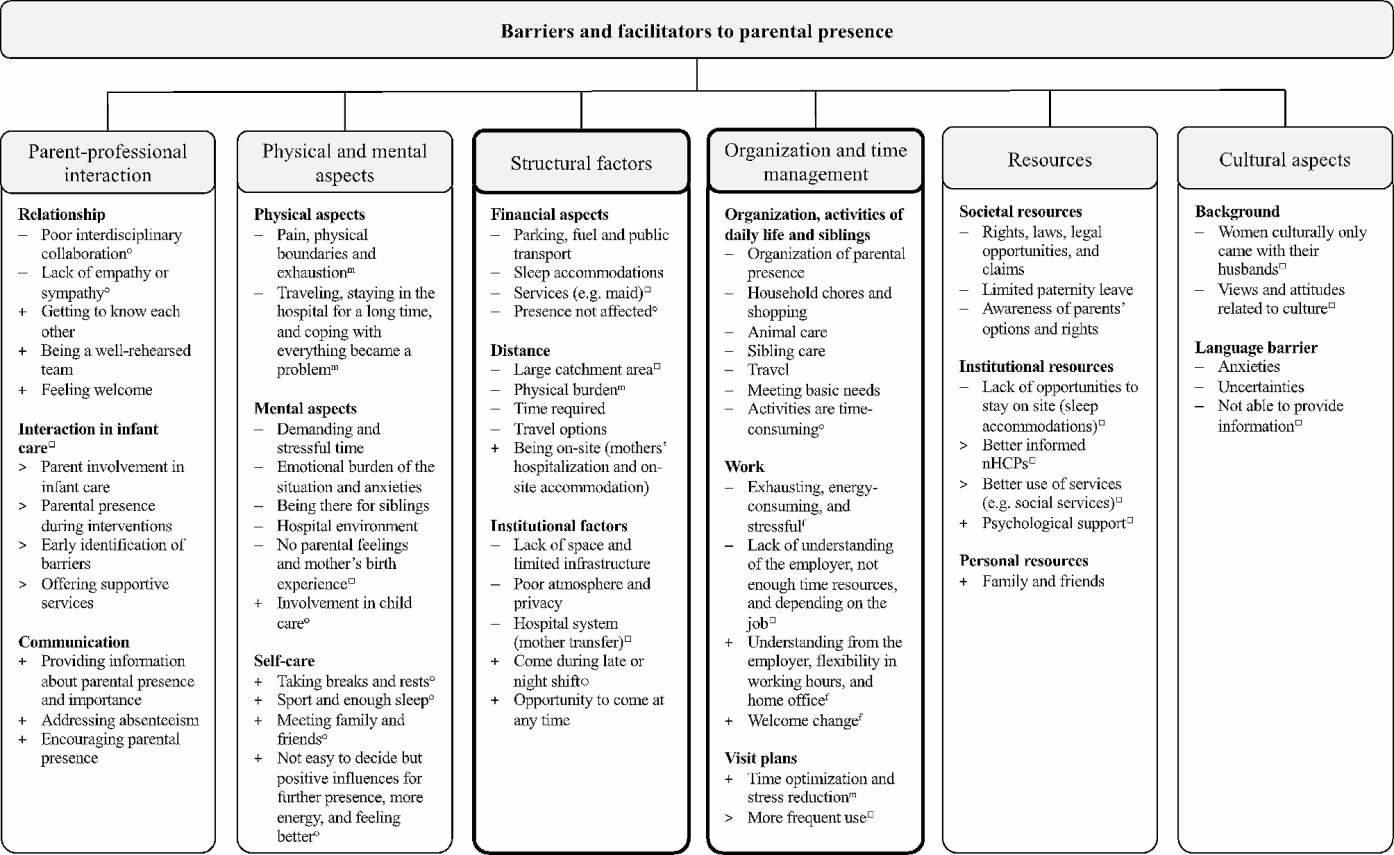
Barriers and facilitators to parental presence. –: barrier; +: facilitator; >: suggestion for improvement; m: affect mothers; f: affect fathers; O: mentioned by parents; :mentioned by nHCPs; KMC: Kangaroo Mother Care; nHCPs: neonatal healthcare professionals

Parental presence showed a wide range of frequency and duration. Most parents reported coming daily, although mothers and fathers differed in the length of stay. Most mothers (M) were present for at least six and up to fourteen hours per day, while fathers (F) came for no more than eight hours, with an average of about three to four hours, mainly depending on their work situation. Six categories of barriers and facilitators to parental presence were explored in the analysis: (1) structural factors, (2) organization and time management, (3) resources, (4) physical and psychological aspects, (5) parent-professional interaction, and (6) cultural aspects (see Fig. 1).

### Structural factors

Parents and nHCPs recognized structural facilitators and barriers to parental presence consisting of financial, travel, and institutional factors. The financial aspects included the high cost of parking, fuel, public transportation, and the cost of the Ronald McDonald House as a place for parents to stay during the infant’s hospitalization.

*“Parking fees, for example, can be a barrier. […] I imagine the Ronald McDonald House costs a lot as well. ” (FGD04, P2, physician)*

Many parents mentioned this as a challenge. However, it did not necessarily affect the frequency or duration of their presence. Physicians mentioned services such as a maid that could reduce the burden on parents but is dependent on funding.

*“Yes, so what I noticed, of course, is the parking. If you go to the hospital parking and you are there all day, you can quickly lose a hundred francs or more a week. […] But other than that, we are not in a bad financial position. […] I think we should make it.” (F08)*

One physician pointed out that the neonatal unit has a large catchment area, resulting in long distances for parents to travel. Parents and nHCPs alike reported travel as a barrier for parents to be present due to the physical burden on mothers, the time required, and travel options. The opposite (short travel), being at the Ronald McDonald House, and being on-site during the mothers’ hospitalization was mentioned as facilitators for parental presence by parents.

*“That was cool, of course, when I was still an inpatient. I went down at nine in the evening, as soon as I could get out again. Or I went down in the morning before breakfast.” (M05)*

Parents and nHCPs experienced the lack of space in the unit, limited infrastructure (few available bonding chairs), crowdy atmosphere, and loss of privacy as making the setting uncomfortable for parents, doing KMC, breastfeeding and long stays. In this regard, a nurse spoke about feedback from fathers that reflect the pleasant and calm atmosphere early on late or night shifts.

*“I’ve also heard from fathers who come early in the morning that the atmosphere in the morning, when there’s just the night guard and all the other kids are asleep, is very nice. Just that quiet.” (FGD02, P5, nurse)*

Neonatal healthcare professionals mentioned the structure of the hospital system as a barrier. Specifically situations where infants were transferred to the neonatal unit of another hospital, but there was no room for the mother to be transferred.

*“And then the mother can’t be transferred because there’s no room for her […]. This is sometimes very difficult to accept, that this transfer of the mother doesn’t seem to be as important as it should be.” (FGD03, P2, physician)*

A positive aspect mentioned by both nHCPs and parents was the opportunity to come at any time without restrictions.

*“So you can really come here whenever you want. That is valuable. For example, I thought that if I could not come during the day, I could come in the evening or at night. You really can always come.” (M01)*

### Organization and time management

Mothers and fathers found it difficult to juggle daily activities and meet their own basic needs during this time. In addition to their daily routines such as shopping, doing household chores, and work, they additionally had to manage animal and sibling care, and travel to the neonatal unit or, to other commitments. Parents described these activities as taking up time, they would otherwise like to spend with their infants. Caring for siblings was difficult to organize, which both parents and nHCPs identified as a barrier. Parents wanted to spend time with all their children, to do them justice, and were often torn when unable to do so.

*“Of course I would have liked to come, but our boy is still at home. I have been in the women’s hospital for so long, even before that. I have to be at home sometime. It is also for him.” (M05)*

Almost every father talked about working full time and missing a lot of time with the infant. They described the infants’ hospitalization combined with their work as exhausting, energy consuming, stressful, and seen as something that could not be changed. One father regarded work as a welcome change and appreciated the social contacts there. In the nHCPs’ perspective, the problem of work as a barrier was in the lack of understanding from the employer, not enough time resources, and dependent on the job where some fathers are more flexible than others.

*“Another thing I hear again and again, for example from fathers, is that the employer may not be so understanding after all.” (FGD01, P3, nurse)*

Fathers found that understanding from the employer, flexibility in working hours, home office, the ability to take leave, and understanding about unexpected short-term absences led to more time with their infants.

*“I usually come in the afternoon or evening. It depends a little bit on whether I have a lot of work to do. If you have a lot of work, that is how it is. I also have to take care of the workplace because my child comes home after. But what else can I do?” (F09)*

Parents and nHCPs experienced visit plans as a supportive tool elaborated in collaboration with an nHCP to structure the days, reducing stress and optimizing time management. Mothers felt more supported than fathers by visit plans that had already been implemented.

*“My wife had a visit plan because of breastfeeding and stuff. However, maybe it would also be good for the fathers if you would support them. Maybe you can come up with some ideas on how to organize things better.” (F10)*

### Resources

Parental presence was identified as dependent on resources and available support at three levels: the societal, the institutional, and the personal level.

For the societal aspect, parents and nHCPs talked about the parents’ legal situation with given rights, laws, legal opportunities and claims. Fathers experienced a legal barrier of having two weeks of paternity leave, which was also mentioned by a nurse as a fundamental problem. Parents and nHCPs also identified difficulties in ensuring parents’ awareness of all their options and rights to get the best support they are entitled to, such as sick leave for childcare or funding from the compensation fund.

*“I also want to give fathers three months, because the first time is the most important. […] The mother has memories with the child, but the father has to work all day and sleep all night. Then it feels like a two-hour visit home. But that’s the law.” (F09)*

The nHCPs summarized missing or underutilized services as part of institutional resources – the lack of opportunities for parents to stay on-site and the lack of space in the Ronald McDonald House, which needs to be improved. In addition to sleep accommodations, they realized that they should be better informed about existing internal and external services, such as social services, childcare, or assistance from an emergency response agency. One physician suggested a guideline with information for parents to help nHCPs counsel and support parents. As another internal example of improvement, they mentioned that social services should be more involved as they can support parents individually in this situation. Psychological support is an already implemented and regularly used service, which benefits parental presence.

*“I think you should also educate yourself a little bit internally about what’s available. So that you know the resources. Maybe there are more than we think. We just don’t know the whole structures enough to offer it optimally.” (FGD04, P4, physician)*

At the personal level, parents and nHCPs recognized family and friends as facilitators through their support in caring for siblings, driving them to the neonatal unit, doing housework, caring for animals, shopping for groceries, and providing emotional support.

*“Yes, immediate family certainly helps. […] They drove me here a lot. Or when they went shopping or when we came back in the evening and were able to sit with them for dinner […]. That made it a lot easier.” (M04)*

### Physical and psychological aspects

Mothers mainly mentioned the physical aspect. They felt worse and had to deal with pain, exhaustion, loss of energy, need for more sleep, and tighter physical boundaries. Because of this condition, traveling to the neonatal unit, staying in the hospital for a long time, and coping with everything around them became a challenge. In addition, the nurses found that the mothers needed more support due to their physical condition, and some of them were reaching their limits.

*“Therefore, my husband was there. Anyone who could stand, or at least sit, could do it. However, that was not possible for me.” (M02)*

Barriers were identified as the emotional burden of the situation itself, experiencing the hospital as a stressful and extremely demanding place, and of caring for and feeling guilty about the siblings. The hospital environment was mainly mentioned by the mothers of the infants with long hospital stays as emotional barrier. In addition to the hospital environment, nHCPs identified anxiety about the preterm infant, lack of parental feelings for the infant, and the mother’s psychological well-being regarding her birth experience as emotional barriers for parents.

*“I sent my husband ahead because I said, “Hey listen, I need to get some fresh air right now. I can’t go in this building just yet.” So I really felt quite bad.” (M01)*

*“You may be afraid of the sick infant and the machinery of neonatology. You might want to look away and protect yourself. Maybe even unconsciously.” (FGD04, P4, physician)*

An important aspect that made the parental presence easier was if the parents practiced self-care. Parents reported taking breaks to rest, do sports, get enough sleep, and to meet with family and friends. It was not easy for them to do so, and they were hesitant to do so. When they did, they reported a positive influence for the rest of their presence, more energy, felt better, and stated that it was critical to avoid any mental breakdowns and to manage forces.

*“I would rather be there all the time. But it’s also good to be able to go home for a while. That is good as well. We also agreed that I would still go to training once or twice a week. Then you can still relax a bit.” (F08)*

*“I would like to be with the children more, but I see that I have to keep my strength in check. […] I have met women who are really with the infants from morning to midnight. If you look at them, it doesn’t take much longer and they collapse.” (M10)*

Another aspect that parents reported as psychologically helpful was being involved in the child’s care, which made them feel like parents.

### Parent-professional interaction

Parents reported poor interdisciplinary collaboration, lack of empathy, and lack of sympathy as negative influences on parental presence. Communicative aspects such as not being taken seriously, being pressured, or having bad conversation experiences also hurt their relationship from the parents’ perspective. In contrast, getting to know each other, being a well-rehearsed team, feeling welcome, or having a good relationship facilitated the parental presence, as reported by parents and nHCPs.

*“Another thing that comes to mind with barriers is how parents feel or how comfortable they feel when they are here […] I think that also makes parents more likely to come or stay longer if they feel comfortable with us.” (FGD03, P4, physician)*

Optimizing care was part of all FGDs on supporting parental presence. They talked about involving parents in the care, letting them take care of the infants, and having parents present during interventions. They emphasized that this depends on the infant’s medical condition and the parents’ willingness, which should always be taken into account. Other optimizations of care were mentioned with early identification of barriers and offering supportive services individually as part of the care process.

Neonatal healthcare professionals reported that providing information about the importance of parental presence, addressing absenteeism, and encouraging parental presence resulted in increased presence. This was also mentioned by mothers and fathers.

*“It would be good if you could be there and do this.” We took that to heart and had to say: “That’s right.” […] That’s certainly why we came more often […] We knew that if we weren’t doing well, we didn’t have to. We have support here. We felt that.” (F05)*

### Cultural aspects

Neonatal healthcare professionals reported that some mothers only came with their husbands to the neonatal unit due to cultural customs. Some experienced the view that the infants were well cared for in the hospital and that parental presence was not needed as related to culture and its different attitudes.

*“Then it depends again, because there are also women who culturally only come with the husband.” (FGD1, P5, nurse)*

Regarding the background, the physicians experienced language barriers. They suspected that this led to anxiety and uncertainty since the parent was not able to ask questions to be properly informed. This was also mentioned by a father who reported that his wife was uncertain about coming to the neonatal unit alone and did not trust her language skills.

*“She doesn’t say what’s on her mind and I think to myself, “Sometimes you just have to speak for yourself.” But she doesn’t dare. The confidence is not there.” (F09)*

## Discussion

Parents and nHCPs experienced barriers and facilitators related to structural factors, organization and time management, activities of daily living and work, societal, institutional, and personal resources, physical and psychological aspects, parent-professional interaction, and cultural background and language. Some perspectives differed between mothers and fathers, while the overall views of parents and nHCPs provided complementary rather than conflicting insights. Our results show that the barriers and facilitators are broadly consistent with findings from other studies of parental presence in FICare interventions.

The structural factors as an outcome rich category of this study are also present in previous studies of various FICare interventions that show similar findings for barriers and facilitators with travel and distance, infrastructure, lack of space, lack of privacy, and capacity of nHCPs [28, 30, 33, 34, 42–44]. Looking at a cross-sectional study from the US, they showed that the structural factor of room type with open bay setting, double occupancy, and single family rooms was a significant predictor of parental presence [26], which may be related to other structural factors such as space or privacy.

Regarding the resources, the most important aspect of our findings investigated in previous studies of FICare interventions and predictors of parental presence is the sleep accommodation as a facilitator to parental presence or implement interventions [28, 30, 36, 37, 43]. A study in six European countries reported that the opportunity to stay overnight was the most important factor in supporting parent-infant closeness [28]. On the other hand, another study on parent-infant closeness reported on the availability of the Ronald McDonald Houses as a possible barrier, because family rooms were earmarked for parents who lived far away [30].

The physical and psychological aspects of parental presence are also represented in studies of KMC, where parents felt anxious about engaging in KMC, experienced the hospital environment as an emotional barrier, and mothers were physically challenged by postpartum pain [33, 34, 42]. Similarly to parents in our study reporting communication, interaction in care, and parent-professional relationship as influencing parental presence, a study on mothers in the US found a lack of support, offer, and information about KMC from nHCPs to negatively impact the use of KMC [42].

The influence of the relationship, good collaboration, and encouragement of nHCPs as facilitators has been found in our study and previous literature on FICare interventions [30, 42, 44]. Concerning these findings of the important interaction between parents and nHCPs, studies of parent-infant closeness and FICare found that educating professionals, acquiring and transferring knowledge skills, and influencing nHCPs’ attitudes were critical for improvement [30, 43].

In addition to numerous congruent and complementary findings, this study presents organization and management as a more recent finding. This category is less common in the literature, with one study of barriers and facilitators to KMC in the US identifying the difficulty of scheduling parental presence around travel, obligations, siblings, and infant feeding times, as well as the work aspect [42]. In addition, one review shows in its findings that an attempt to increase parental presence by scheduling weekly appointments was successful in increasing maternal presence [36]. As in this study, the aforementioned study did not report on the application and effects on the father, which underlies the suggestion for improvement.

Our study collected data on three perspectives: mothers, fathers and nHCPs. Given that mothers and fathers are affected by different aspects is essential to provide insight on the respective perspectives. Mothers were more affected by physical and organizational aspects related to on-site activities such as breastfeeding, while fathers were mainly affected by aspects related to work such as short paternity leave and managing life besides hospitalization. In a study of the implementation of FICare interventions in Sweden, Norway, and The Netherlands, fathers were also affected by the need to return to work and not being able to be present [43]. Another study interviewed mothers about influences on KMC and referred to the physical burden after birth [42]. Also important are the perspectives of both parents and nHCPs, who did not disagree but reported different and complementary aspects. Parents in our study were able to give more insight into their experiences and feelings in terms of physical and mental aspects, organization, and time management in terms of which activities affect them, and the impact of parent-professional interaction. Neonatal healthcare professionals’ perspectives focus more on institutional aspects such as structural factors with limited space, transfer of mothers as barriers, available internal and external services, and the need to improve regarding the use of these services and that nHCPs should be better informed. A study on KMC also interviewed both, which helped to get a comprehensive understanding with e.g., nHCPs bringing in the medical benefits and issues on barriers and facilitators to perform KMC [34].

### Strengths and limitations

A strength of the study is the inclusion of the three perspectives of mothers, fathers, and nHCPs to gain a comprehensive understanding of barriers and facilitators of the individuals directly involved in this specific setting. Moreover, this study is the first to provide insights into the barriers and facilitators of parental presence in the neonatal unit in the Swiss hospital system. One limitation of the study is the inclusion of only German-speaking parents to ensure in-depth understanding and accurate extraction of data in the qualitative interviews. This may influence the findings regarding cultural and financial aspects, and parental resources concerning refugees or foreigners living in Switzerland with no or a smaller social environment. More comprehensive data may be obtained by including more languages and thus more cultural diversity in future research. Furthermore, this study only reflects the barriers and facilitators experienced regarding only one of the neonatal units in Switzerland. Another limitation is the lack of member check, which affects the trustworthiness of the study and creates a risk of misunderstanding.

## Conclusions

Multifactorial barriers and facilitators determine parental presence and experience in the neonatal unit. In this study, parents and nHCPs made specific recommendations to improve parental presence. Further research on this topic in Switzerland and other countries would provide a more complete picture of the issue.

### Electronic supplementary material

Below is the link to the electronic supplementary material.

Supplementary Material 1

Supplementary Material 2

## Data Availability

No datasets were generated or analysed during the current study.

## Abbreviations

F: Father
FGD: Focus group discussion
FICare: Family Integrated Care
KMC: Kangaroo Mother Care
M: Mother
nHCP: Neonatal healthcare professional
P: Participant
SD: Standard deviation
