# Supplementary material for: Parents’ and neonatal healthcare professionals’ views on barriers and facilitators to parental presence in the neonatal unit: a qualitative study
Source: BMC Pediatr. 2024 Apr 24;24:268. doi: 10.1186/s12887-024-04758-3 (PMC11040849; doi:10.1186/s12887-024-04758-3)
Supplement: Supplementary file 1 — Supplementary Material 1 [file 12887_2024_4758_MOESM1_ESM.docx]

**Additional file 1. Interview guide**

**Introduction**

1. Interviewer introduces herself
2. Interview procedure and key data

- Duration: about 20 minutes
- First, I will ask you questions about yourself and then we will talk about the time you spent with your infant in the neonatal unit.
- The interview will be recorded, transcribed and analyzed. As described in the information for parents, all data will be anonymized. No data/statement can be traced back to you or your infant.
- You do not have to answer questions if you do not want to. Questions may remain unanswered.

Do you have any questions before we start?

**Characteristics**

1. Parent:

Mother Father

1. Age (in years)

<20 20–25 26–29 30–34 > 35

1. Nationality

Switzerland

If not: nationality, length of stay in Switzerland and identity card status: ____________________

1. Number of children

1 child 2 children 3 children >4 children

1. Do you consent to the use of your child's previous inpatient treatment and care, birth mode, and other key birth data from the documentation system for the study?

Yes No

Birth mode:

Spontaneous birth Vaginal operative birth (vacuum or forceps)

Caesarean section: primary secondary

Child's gestational age at birth (in weeks of pregnancy)

< 29 ^6/7^ 30 ^0/7^–32 ^6/7^ 33 ^0/7^–34 ^6/7^ 35 ^0/7^–36 ^6/7^

> 37

Your child's previous length of stay in the neonatal unit (in days): _______________

**Interview guide**

| **Opening question:** How do you feel about the time you spend with your infant in the neonatal unit? | | |
| --- | --- | --- |
|  | **Key questions** | **Follow-up questions** |
| Frequency  and  Duration | - How would you describe the frequency and duration of your presence? - How often and how long per week can you spend time with your infant? | - Why do you come to the neonatal unit? - Are there any reasons why you do not come? If so, why? - How often or how long do you think parents should be present in the neonatal unit? |
| Satisfaction | - How satisfied are you with the frequency and length of time you spend with your infant? | - Why are you not happy? - How does it affect you when you can't be with your infant, or for less time than you'd like? - Do you think you are there often enough? - What would be your ideal day in the neonatal unit? |
| Barriers  and  facilitators | - What barriers do you face in spending time with your child? - What challenges do you face when you come to the neonatal unit? - What makes it easier for you to be in the neonatal unit? - What helps you spend time with your infant? | - How did you come up with this barrier/facilitator? - What would be an example of such a barrier/facilitator? - Why does it affect your behavior? - What do you think causes this barrier/facilitator? - What would help you deal with this barrier? |
|  |  | Literature research topics (questions after free narration!)   - Child care, financial barriers, transportation and time - Emotional: anxiety/physical postpartum recovery, aftermath of birth - Professionals: absence, lack of information, support |
| Are there any issues or concerns that are important to you that we have not discussed? | | |
| V4.29.04.2022 | | |
