# Supplementary material for: Parents’ and neonatal healthcare professionals’ views on barriers and facilitators to parental presence in the neonatal unit: a qualitative study
Source: BMC Pediatr. 2024 Apr 24;24:268. doi: 10.1186/s12887-024-04758-3 (PMC11040849; doi:10.1186/s12887-024-04758-3)
Supplement: Supplementary file 2 — Supplementary Material 2 [file 12887_2024_4758_MOESM2_ESM.docx]

**Additional file 2. Focus group discussion guide**

**Introduction**

1. Interviewer introduces herself
2. Discussion procedure and key data

- Duration: Max. 30 minutes
- Guided group discussion on the topic "Facilitators and barriers to parental presence in neonatal unit".
- The discussion will be recorded for later transcription and analysis. All data will be anonymized and no data or statements will be traceable to you.
- Important aspects of the discussion:
- We let each other speak.
- Differences of opinion can be discussed and are accepted.
- The interview will remain confidential.

**Focus group discussion guide**

| **Opening question:**  What comes to your mind when you hear the topic "barriers and facilitators to parental presence in the neonatal unit"? | | |
| --- | --- | --- |
| **Key questions** | **Follow-up questions** | **Literature research topics** |
| What can you say about the importance of parental presence in the neonatal unit? | - Why do you think parental presence is (not) important? - How do you perceive the importance of parental presence? - Do you make parents aware of its importance? If so, how? | FICare: KMC, skin-to-skin contact  Strengthening parent-infant bonding  Positive effects for the infant: improvement in cardiac and respiratory outcomes  Parental benefits: resilience |
| What differences do you experience with parental presence? | - How often and for how long should parents be present? - What are your recommendations? - Do you share your recommendations with parents? If so, how? |  |
| How satisfied are parents with the frequency and length of time they spend with their infant in the neonatal unit? | - When are parents satisfied? - Do you think parents are present often enough? - What situations of dissatisfaction have you experienced? |  |
| What barriers do you think parents face in spending time with their infant in the neonatal unit?  What do you think, does the presence of the parents facilitate or support? | - What are your experiences with barriers/facilitators to parental presence? - What is the most common barrier for parents? - Why is it a barrier/facilitator? - What barrier/facilitator situations have you experienced? - Are there times when you find parents annoying? - What would be an ideal day for parents? | Emotional, physical, and structural barriers  Child care  Emotional stress: anxiety  Physical stress: postnatal recovery, consequences of childbirth  Financial barriers  Transportation and time  Professionals: absence, lack of information, support |
| Do you see any adjustments that could be made to optimize the opportunities for parental presence? | - What do you think could support parents during their time with their infants? - What would you recommend to parents to support them in their time with their infants? | Environmental adaptations  Child Care  Restrictions imposed by institutions ("visiting hours") |
| Are there any issues or concerns that you feel are important that we have not discussed? | | |

V4.29.04.2022
